# Draft Genome Sequence of *Thermoanaerobacter* sp. Strain A7A, Reconstructed from a Metagenome Obtained from a High-Temperature Hydrocarbon Reservoir in the Bass Strait, Australia

**DOI:** 10.1128/genomeA.00701-13

**Published:** 2013-09-12

**Authors:** Dongmei Li, Paul Greenfield, Carly P. Rosewarne, David J. Midgley

**Affiliations:** CSIRO Animal, Food and Health Sciences, North Ryde, NSW, Australiaa; CSIRO Mathematics, Informatics and Statistics, North Ryde, NSW, Australiab

## Abstract

The draft genome sequence of *Thermoanaerobacter* sp. strain A7A was reconstructed from a metagenome of a microbial consortium obtained from the Tuna oil field in the Gippsland Basin, Australia. The organism is a strict anaerobe that is predicted to ferment a range of simple sugars and undertake sulfur reduction.

## GENOME ANNOUNCEMENT

In 2011, fluid samples were collected from the A7A oil well in the Tuna oil field (38°10′S, 148°25′E) in the Gippsland Basin, Australia. Chemical analyses of the water revealed the sample was pH 7.2, had a salinity of 2.7%, and contained 270 mg liter^–1^ calcium, 320 mg liter^–1^ magnesium, 1,200 mg liter^–1^ sulfate, and 1,170 mg liter^–1^ bicarbonate. The temperature in the reservoir was 102°C. One liter of formation water was filtered through a 0.2-µM polyvinylidene difluoride (PVDF) filter disc and subjected to genomic DNA extraction using a Meta-G-Nome DNA isolation kit (Epicenter Biotechnologies). The resultant DNA was sequenced using 100-bp paired-end Illumina HiSeq at the University of Western Sydney. A genome was separated from the metagenome and assembled using the procedure described by Sutcliffe et al. (2013) (1). This approach identified three abundant organisms in the metagenome sample: *Thermotoga maritima* A7A (1), *Desulfonauticus* sp. strain A7A, and *Thermoanaerobacter* sp. strain A7A. The latter is the subject of this genome announcement.

The draft genome sequence of *Thermoanaerobacter* sp. A7A is 2,614,610 bp in length and comprised 178 large contigs (>1,000 bp). These contigs ranged in size from 1,012 bp to 130,463 bp, with mean and median contig lengths of 14,689 and 5,890 bp, respectively. The mean GC content of the genome was 34.1%. Annotation was performed using Integrated Microbial Genomes Expert Review (IMG ER) (2), which predicted a total of 2,797 protein-coding genes.

Cultured isolates of *Thermoanaerobacter* species are strictly anaerobic, thermophilic-to-hyperthermophilic bacteria of the *Firmicutes* family *Thermoanaerobacteriaceae* (3–5). *Thermoanaerobacter* sp. A7A appears, based on 16S rRNA gene identity (>99%), to be most closely related to *Thermoanaerobacter brockii* subsp. *finnii*, isolated from a high-temperature (92°C) oil field (5). Like *T. brockii*, *Thermoanaerobacter* sp. A7A appears to be a hyperthermophilic anaerobe.

Annotation of the genome of *Thermoanaerobacter* sp. A7A suggests that the organism is capable of NADPH-dependent sulfur reduction to hydrogen sulfide. Other *Thermoanaerobacter* species can ferment a range of simple hexose and pentose sugars (3–5), and *Thermoanaerobacter* sp. A7A appears to be similar. The annotation of the draft genome sequence indicates the presence of genes that likely facilitate growth on monosaccharides (arabinose, glucose, fructose, and xylose) and disaccharides (cellobiose, maltose, and sucrose), along with the degradation of more complex polysaccharides, including starch. The genome does appear to encode a cellulase; however, most *Thermoanaerobacter* species examined to date are noncellulolytic. The cellulose-degrading potential of *Thermoanaerobacter* sp. A7A remains unclear.

The draft genome sequence shows that this organism has a host of genes involved in flagellum formation, including *flgD*, *flgC*, *flgL*, *flgK*, *flgG*, *flgS*, and *flgM*, and is probably peritrichous, like most *Thermoanaerobacter* species (4). It also has genes involved in chemotaxis/flagellar motation components, including *motA* and *motB*.

The other two species that occur in the Tuna oil field at the A7A oil well, *Thermotoga maritima* A7A (1) and *Desulfonauticus* sp. A7A, will be described in separate genome announcements and a comparative analysis will be undertaken to understand how these three species interact in the subsurface.

### Nucleotide sequence accession number.

This whole-genome shotgun project has been deposited at DDBJ/EMBL/GenBank under the accession number AVAF00000000. The version described in this paper is the first version.
